# Factors Associated With New‐Onset Depression Following Ischemic Stroke: The Women's Health Initiative

**DOI:** 10.1161/JAHA.116.003828

**Published:** 2017-02-02

**Authors:** Joel Salinas, Roberta M. Ray, Rami Nassir, Kamakshi Lakshminarayan, Christina Dording, Jordan Smoller, Sylvia Wassertheil‐Smoller, Jonathan Rosand, Erin C. Dunn, Jacques Rossouw, Shari Ludlam, Dale Burwen, Joan McGowan, Leslie Ford, Nancy Geller, Garnet Anderson, Ross Prentice, Andrea LaCroix, Charles Kooperberg, JoAnn E. Manson, Barbara V. Howard, Marcia L. Stefanick, Rebecca Jackson, Cynthia A. Thomson, Jean Wactawski‐Wende, Marian Limacher, Robert Wallace, Lewis Kuller, Sally Shumaker

**Affiliations:** ^1^ Department of Neurology Massachusetts General Hospital Harvard Medical School Boston MA; ^2^ Department of Psychiatry Massachusetts General Hospital Harvard Medical School Boston MA; ^3^ Center for Human Genetics Research Massachusetts General Hospital Harvard Medical School Boston MA; ^4^ Division of Public Health Sciences Fred Hutchinson Cancer Research Center Seattle WA; ^5^ Department of Biochemistry and Molecular Medicine University of California Davis CA; ^6^ Division of Epidemiology and Community Health School of Public Health University of Minnesota Minneapolis MN; ^7^ Stanley Center for Psychiatric Research The Broad Institute of Harvard and MIT Cambridge MA; ^8^ Department of Epidemiology and Population Health Albert Einstein College of Medicine Bronx NY

**Keywords:** epidemiology, risk factor, stroke, women, Epidemiology, Risk Factors, Women, Ischemic Stroke, Quality and Outcomes

## Abstract

**Background:**

Psychosocial characteristics have a strong effect on risk of depression, and their direct treatment with behavioral interventions reduces rates of depression. Because new‐onset poststroke depression (NPSD) is frequent, devastating, and often treatment‐resistant, novel preventive efforts are needed. As a first step toward developing behavioral interventions for NPSD, we investigated whether prestroke psychosocial factors influenced rates of NPSD in a manner similar to the general population.

**Methods and Results:**

Using the Women's Health Initiative, we analyzed 1424 respondents who were stroke‐free at enrollment and had no self‐reported history of depression from enrollment to their nonfatal ischemic stroke based on initiation of treatment for depression or the Burnam screening instrument for detecting depressive disorders. NPSD was assessed using the same method during the 5‐year poststroke period. Logistic regression provided odds ratios of NPSD controlling for multiple covariates. NPSD occurred in 21.4% (305/1424) of the analytic cohort and varied by stroke severity as measured by the Glasgow scale, ranging from 16.7% of those with good recovery to 31.6% of those severely disabled. Women with total anterior circulation infarction had the highest level (31.4%) of NPSD while those with lacunar infarction had the lowest (16.1%). Prestroke psychosocial measures had different associations with NPSD depending on functional recovery of the individual.

**Conclusions:**

There is a difference in the relationship of prestroke psychosocial status and risk of NPSD depending on stroke severity; thus it may be that the same preventive interventions might not work for all stroke patients. One size does not fit all.

## Introduction

Depression after stroke affects ≈1 in every 3 to 5 stroke survivors within the first 5 years after stroke^1^ and is closely associated with impaired recovery^2^ and increased long‐term morbidity and mortality.^3^, ^4^ Despite more frequent use of antidepressants after stroke,^5^ there has yet to be a decrease in the prevalence of poststroke depression.^1^ These findings underscore that current treatments for depression are only modestly effective; limited treatment efficacy may be due to multiple factors (eg, frequency, duration, dose, and specific method of treatment). Indeed, large‐scale systematic reviews on the efficacy of treatment for depression after stroke have shown that antidepressants may reduce depressive symptoms, albeit with increased side effects; meanwhile, the evidence is variable for benefits from nonpharmacological interventions such as psychotherapy.^6^ Current treatments for poststroke depression are inadequate, but given existing evidence for benefit from *early* psychological therapy in improving mood and preventing depression,^7^ the possibility remains that prevention of poststroke depression may be a more effective approach than treating depression once it has emerged.^8^, ^9^

Predicting which patients are at highest risk for developing poststroke depression at the time of stroke is critical to determining whether early pharmacologic or psychosocial interventions can prevent stroke‐related depression. However, proposed risk and protective factors of poststroke depression only partly explain observed variation in risk.^10^, ^11^ Therefore, a greater understanding of prestroke risk factors for *new‐onset poststroke depression* (NPSD), meaning first occurrence of depression after stroke in the absence of recent depression history before the stroke, is needed to isolate predisposing factors for depression that are specific to the unique psychosocial and neurophysiologic milieu of stroke as opposed to longstanding depression from factors unrelated to stroke. The study of prestroke risk factors for NPSD may be a critical missing piece, as exploration of these factors may help clarify specific risks in the setting of stroke that may be most responsive to early empiric antidepressant use or behavioral interventions^12^ immediately after stroke to prevent development of poststroke depression.

Accumulating evidence in depression more generally supports a central role for psychosocial factors, such as persistent negative affect and optimism (the expectation that positive events will occur in the future),^13^ in influencing cardiovascular disease outcomes.^14^, ^15^ However, only 3 prospective studies of stroke cohorts^16^, ^17^, ^18^ have examined the relationships between psychosocial factors and poststroke depression.^10^, ^19^ These studies were small, did not reliably collect prestroke data, and only examined general personality traits and composite measures of social support.

To improve ascertainment of stroke survivors at risk for depression who may benefit from early prevention in the acute to subacute poststroke period, a longitudinal cohort of stroke survivors followed prior to their stroke is needed to (1) estimate the odds of poststroke depression in the absence of depressive symptoms *before* the stroke, and (2) identify prestroke psychosocial factors associated with the risk for developing new‐onset poststroke depression.

## Methods

### Study Sample

We identified 1424 women without a baseline history of stroke or depression and who subsequently were diagnosed with an ischemic stroke out of a total 161 808 participants from the Women's Health Initiative (WHI), the largest existing, population‐based, epidemiological cohort that includes prospective study of health associations with psychosocial factors in postmenopausal women ages 50 to 70 in the United States (www.whi.org). As described elsewhere,^20^ this multicenter study consists of the WHI Observational Study (n=93 676) and 3 overlapping randomized clinical trials (CT; n=68 132). Exclusion criteria for enrollment were involvement in other randomized trials, presence of risk factors that would impair participation in the study protocols (eg, active alcoholism, drug dependency, dependence in activities of daily living, dementia, or diagnosed mental illness including active severe depression), or a high likelihood of death or relocation within 3 years after enrollment.^21^ Enrollment and data collection began in 1993–1998 and the cohorts have been followed through the main WHI study (1993–2005), Extension 1 (2005–2010), and Extension 2 (2010–2015). Follow‐up data were collected through August 29, 2014. The current analysis included participants with incident strokes through Extension 1 given that most Extension 2 stroke participants did not have an opportunity to be assessed for depression after stroke. Each study center obtained informed consent and institutional review board approval.

Six thousand four hundred eighteen women experienced a first‐time stroke between enrollment and 2014. Our analyses focused on the 1424 women who were stroke free at enrollment, had no antidepressant use or self‐reported depression at enrollment, experienced a nonfatal or nonvegetative ischemic stroke during the main WHI or Extension 1 study period, and did not develop depression in the timeframe between enrollment and the incident stroke (Figure). Given the heterogeneity and markedly different nature of hemorrhagic stroke management and impact on outcomes compared to ischemic stroke, our study focused on ischemic stroke. Among the 1424, 207 participated only in the main WHI, 332 participated in the main WHI and Extension 1, and 885 participated through Extension 2; 54% were enrolled in CT (v. WHI Observational Study).

**Figure 1:**
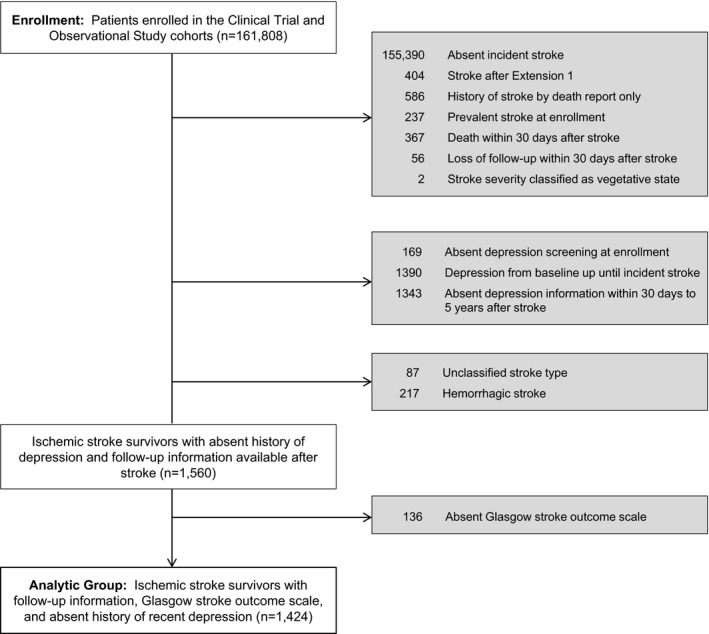
Derivation of the analytic sample.

### Stroke Assessment

New diagnosis of stroke was assessed annually with WHI neurologists adjudicating all potential strokes using standardized study criteria.^20^ Transient ischemic events and all events not requiring hospitalization were excluded. Stroke was classified by brain imaging as follows: *ischemic* (rapid onset of neurologic deficit lasting greater than 24 hours due to an occlusion of cerebral or precerebral arteries with infarction without evidence of other etiology), *hemorrhagic* (based on subarachnoid or intraparenchymal blood on computed tomography or magnetic resonance imaging), or undefined (met criteria for stroke but did not have adequate information for categorization). Stroke subtypes were categorized by vascular territory using Oxfordshire Community Stroke Project classification: total anterior circulation infarct, lacunar infarct, partial anterior circulation infarct, or posterior circulation infarct.^22^ Information regarding stroke location was not available for analysis. Stroke severity was determined using functional recovery at time of discharge as measured by Glasgow Stroke Outcomes Scale scores from hospital records and categorized as good recovery (able to return to work or school), moderate disability (able to live independently, unable to return to work or school), severe disability (able to follow commands, unable to live independently), vegetative survival, death, or unable to assess.

### Depression Assessment

We defined depression using 2 data sources consistent with extant WHI studies.^23^, ^24^, ^25^ First, depressive symptoms were evaluated using the 8‐item Burnam screening instrument, which combines, in a linear regression model,^26^ 6 items from the Center for Epidemiologic Studies Depression Scale 27 and 2 items from the Diagnostic Interview Schedule.^28^ A score of 5 or greater (out of 18) corresponds to a cut point of 16 (out of 60) in the full 20‐item Center for Epidemiologic Studies Depression Scale scale.^26^ The 6‐item Center for Epidemiologic Studies Depression Scale scale correlates highly with the full 20‐item scale (*r*=0.88),^29^ which is commonly used to measure depressive symptoms in epidemiological samples and has been validated among patients with stroke.^30^ For the 6 Center for Epidemiologic Studies Depression Scale items, patients were asked to rate how often (0=rarely or none of the time; 3=most or all of the time) they experienced core symptoms of depression during the past week: felt depressed; sleep was restless; enjoyed life; had crying spells; felt sad; felt that people disliked you. For the Diagnostic Interview Schedule, the 2 validated items asked “In the past year, have you had 2 weeks or more during which you felt sad, blue, or depressed or lost pleasure in things that you usually cared about or enjoyed?” and “Have you had 2 years or more in your life when you felt depressed or sad most days, even if you felt okay sometimes? If yes, have you felt depressed or sad much of the time in the past year?” (1 point for each if yes).^26^

While the Burnam screen does not independently diagnose major depressive disorder and may capture symptoms associated with anxiety or psychological distress, prior studies in the WHI^31^ and elsewhere^26^ have shown the 0.06 cutoff point has 74% sensitivity and 87% specificity for detecting past‐month depressive disorder as diagnosed by psychiatrist‐administered semistructured clinical interviews using the *DSM‐IV* (*Diagnostic and Statistical Manual of Mental Disorders, Fourth Edition*). The Burnam screen has a negative predictive value of 99% despite a positive predictive value of 20% and overall error rate of 14%,^31^ which may be indicative that, as a screening instrument, it may also reflect anxiety, psychological distress, or subsyndromal symptoms; thus in the clinical context this would still prompt referral for additional psychiatric evaluation. Therefore, participants with a Burnam score of 0.06 or greater, for notational convenience, are described interchangeably as having current *depression* or high depressive symptomatology.^29^

The second data source was the initiation of treatment for depression, defined as new antidepressant medication use within 5 years after incident stroke, given that medication information was collected at more frequent intervals than Burnam assessments. This case definition is consistent with previous WHI studies of depression showing both positive and null relationships between exposure groups of interest.^24^, ^25^

With regard to the schedule for depression assessments, at minimum the Burnam assessment was collected at enrollment, first follow‐up visit, and closeout for CT participants, and at enrollment and Year 3 for WHI Observational Study participants. One additional Burnam assessment was performed on all women who continued follow‐up into Extension 2. During main WHI Years 3, 6, and 9, a subset of CT participants had supplementary Burnam assessments. Throughout the main WHI period, women were also asked to bring all current medications in their original pill bottles to their baseline and follow‐up clinic visits (for WHI Observational Study, main WHI Year 3; for CT, main WHI Years 1, 3, 6, and 9). WHI staff recorded all label information, including names of medications used regularly (more than 2 weeks), dose, and duration of use. Starting at Extension 1, medication information was updated using a standardized medical update form. Medication information was then entered into the pharmacy database (Master Drug Data Base; Medi‐Span, Indianapolis, IN). Participants were also asked during Extension 1, as part of a yearly medical update, whether a physician had ever prescribed treatment for depression (pills or therapy) for the first time. We combined data from the medical update with the pharmacy database to determine initiation of “treatment for depression” (including new use of selective serotonin reuptake inhibitors, monoamine oxidase inhibitors, tricyclics, modified cyclic agents, or other medications classified as antidepressants).^23^ Earliest initiation date of antidepressants was determined using the reported duration of treatment use or, if not provided, the date her form was completed.

### Psychosocial Variables

We explored the relationship between NPSD (outcome) and the following *prestroke* psychosocial factors (exposures) measured at the examination closest to the time of incident stroke. These constructs were selected based on previous hospital‐ and rehabilitation‐based studies suggesting their potential association with risk of depression after stroke.^10^ *Optimism* was assessed using 6 items from the Life Orientation Test‐Revised (LOT‐R)^13^ where participants indicated to what extent they agreed (ie, strongly disagree to strongly agree) with statements such as “in unclear times, I usually expect the best” and “if something can go wrong for me, it will.” Summed scores ranged from 6 to 30 where higher scores indicated greater optimism. *Hostility* (ie, dislike and distrust of others) was measured with the cynical hostility subscale of the Cook‐Medley Questionnaire.^32^ This subscale contained 13 true/false items with a possible total range of 0 to 13 with higher scores indicating greater hostility. Sample items include, “no one cares much about what happens to you” and “it is safer to trust nobody.” *Adverse life events*, or indicators of life stress, were captured using modified measures from the Alameda County Epidemiology Study.^33^ Summed scores ranged from 0 to 33, where higher scores indicated participants experienced a greater number of more upsetting events weighted by the participant's judgment of upset due to the event. Sample items included, “Were you physically abused by being hit, slapped, pushed, shoved, punched, or threatened with a weapon by a family member or close friend?” and “Did you have any major accidents, disasters, muggings, unwanted sexual experiences, robberies, or similar events?” *Social support* was measured using a total score constructed from social support subscales from the Medical Outcomes Study questionnaire.^34^ The questionnaire was designed to measure the amount of social support available to participants, with 9 items asking participants to indicate how often different kinds of support were available to them. Total summed scores ranged from 9 to 45, with higher scores indicating greater support. Support subscales included *affection* (“someone to love you and make you feel wanted”), *tangible* (“someone to help you with daily chores if you are sick”), *emotional/information* (“someone you can count on to listen to you when you need to talk”), and *positive social interactions* (“someone to have a good time with”).

### Covariates

Covariates were selected a priori based on established associations in previous WHI studies. *Demographic* covariates included the following: age at time of stroke, self‐reported race/ethnicity, highest level of education (college degree or greater), WHI study cohort, and a normalized neighborhood socioeconomic scale index.^35^ *Behavioral* covariates included: smoking status (current or past smoker),^36^ alcohol intake (nondrinker, past drinker, <1 drink per month, <1 drink per week, 1 to <7 drinks per week, 7 or more drinks per week),^29^ and lack of exercise (no episodes per week of moderate or strenuous activity ≥20 minutes).^29^ *Health‐related* covariates included: obesity (from previous study,^37^ body mass index >27.3 kg/m^2^), and waist circumference (cm).^37^ *Comorbid* covariates included: treatment of diabetes mellitus (oral or injection),^38^ treatment of elevated cholesterol,^29^ treatment of hypertension or hypertension status (systolic blood pressure ≥140 mm Hg and/or diastolic blood pressure ≥90 mm Hg),^29^ and active hormone use at enrollment (unopposed estrogen and estrogen with progesterone).^39^

### Statistical Analysis

We examined unadjusted 5‐year NPSD occurrence by stroke subtype, stroke severity, and prestroke psychosocial factors as well as demographic, behavioral, health‐related, and comorbid characteristics. Given consistent associations between stroke severity (more so than stroke laterality) and risk of poststroke depression,^10^ we conducted a set of logistic regression models stratified by stroke severity (good recovery versus moderate to severe disability) to examine the association between each psychosocial exposure and odds of developing NPSD within 5 years after stroke. We examined correlations (Pearson for categorical and polyserial for continuous) to evaluate the bivariate association between our exposure variables and identify any pairs of covariates that were too highly correlated and should be excluded from the analysis to reduce the likelihood of multicollinearity. We also included covariates in our models based on a priori knowledge that were likely be associated with both outcome and exposure but not on the causal pathway between exposure and outcome. We additionally assessed for model stability by performing 3 regression models for each stroke severity substrata as follows: (1) adjusted by age at stroke and race (white versus nonwhite); (2) additional adjustment for college education and normalized neighborhood socioeconomic status index; and (3) additional adjustment for a priori potential confounders. For sensitivity analyses, we performed the 3 regression models using NPSD cases defined only by the Burnam depression screen to assess for internal validity of our results by excluding cases defined by antidepressant use. Given our exclusion of women who were classified as having prestroke depression from our analytic sample to select only those at risk of new‐onset depression after stroke, we conducted a post hoc analysis inclusive of women who had depression during the 5 years prior to stroke. Furthermore, to assess for the possibility that a depressed participant may be more likely to miss a study activity—thus biasing results if depressed patients were misclassified—we compared the ratio of completed versus scheduled study activities where women had the opportunity to report depression information by the following categories: *prestroke* depression status, *poststroke* depression status, and psychosocial exposure variables.

Statistical significance for the regression models was set to a 2‐sided α≤0.05. We present results as odds ratios (OR) and Wald 95% CIs. Power calculations suggested that in the fully adjusted models there would be at least 80% power to detect small effects of exposure (ie, OR effect size of 0.50) for NPSD at a 2‐tailed significance of 0.05.^40^ All analyses were conducted in SAS version 9.4 (SAS Institute, Inc, Cary, NC).

## Results

New‐onset depression after ischemic stroke occurred in 21.4% (305/1424) of the analytic sample (Table 1). Among cases, 41 were determined only by Burnam, 251 only by initiation of treatment for depression, and 13 by both. NPSD varied by stroke severity ranging from 16.7% (125/748) of those with good recovery to 31.6% (78/247) of those with severe disability. Stroke severity at the time of hospital discharge was highly correlated with physical functioning^41^ at the time of depression assessment (Spearman correlation coefficient, *r*=−0.17; *P*<0.001). Participants with total anterior circulation infarction had the highest proportion of NPSD (31.4%, 11/35), while those with lacunar infarction (16.1%, 66/411) had the lowest. The distribution of Oxfordshire classifications among cases was similar when the 136 women without stroke severity information were included.

**Table 1:** Characteristics of 1424 Postmenopausal Women With Ischemic Stroke, Self‐Reported Absence of Depression Before Stroke, and Available Stroke Severity and Depression Information Within 5 Years After Stroke

	N (%)	N (%)
	Absence of New‐Onset Poststroke Depression	New‐Onset Poststroke Depression
Total	1119 (79)	305 (21)
Age at stroke, y		
<70	229 (78)	63 (22)
70 to 74	272 (82)	58 (18)
75 to 79	315 (77)	96 (23)
≥80	303 (77)	88 (23)
Race/ethnicity		
White	951 (78)	271 (22)
Black	102 (80)	25 (20)
Hispanic/Latina	13 (87)	2 (13)
Other and unspecified	53 (88)	7 (12)
College education		
Less than college degree	708 (79)	185 (21)
College degree or higher	404 (78)	117 (23)
Normalized neighborhood socioeconomic status index		
Low (<72.0)	245 (77)	75 (23)
Moderate (72.0–77.0)	261 (80)	66 (20)
High (77.1–81.1)	252 (77)	76 (23)
Very high (≥81.2)	257 (81)	62 (19)
Diabetes mellitus^a^		
No	1037 (78)	286 (22)
Yes	80 (81)	19 (19)
Hypertension^b^		
No	486 (79)	126 (21)
Yes	633 (78)	179 (22)
Hyperlipidemia^c^		
No	875 (78)	243 (22)
Yes	160 (81)	37 (19)
Hormone use^d^		
No	387 (81)	92 (19)
Yes	710 (78)	206 (23)
Waist circumference, cm		
Low (<78.5)	278 (79)	73 (21)
Moderate (78.5–86.4)	263 (77)	78 (23)
High (86.5–95.9)	292 (79)	76 (22)
Very high (≥96.0)	280 (78)	78 (22)
Current or past smoker		
No	581 (79)	155 (21)
Yes	525 (79)	143 (21)
Alcohol consumption		
Nondrinker	142 (81)	33 (11)
Past drinker	218 (82)	47 (16)
<1 drink per month	133 (79)	35 (12)
<1 drink per week	222 (75)	75 (25)
1 to <7 drinks per week	252 (79)	68 (23)
7 drinks or more per week	146 (77)	43 (14)
Physical activity^e^		
No activity	176 (80)	43 (20)
Some activity	461 (79)	126 (22)
2 to 4 episodes per week	168 (78)	48 (22)
≥4 episodes per week	241 (78)	67 (22)
Optimism		
Low (<22)	239 (74)	82 (26)
Moderate (22–23)	269 (81)	63 (19)
High (24–25)	283 (79)	75 (21)
Very high (≥26)	310 (79)	83 (21)
Hostility		
Low (0–1)	306 (80)	76 (20)
Moderate (2–3)	289 (81)	69 (19)
High (4–5)	251 (76)	78 (24)
Very high (≥6)	246 (78)	71 (22)
Adverse life events		
None	318 (77)	94 (23)
Low (1–2)	314 (82)	67 (18)
Moderate (3–4)	271 (78)	75 (22)
High (≥5)	211 (76)	67 (24)
Overall social support		
Low (<33)	250 (78)	72 (22)
Moderate (33–37)	273 (82)	58 (18)
High (38–42)	264 (77)	79 (23)
Very high (≥43)	315 (77)	92 (23)
Emotional/informational support		
Low (<17)	592 (81)	141 (19)
High (≥17)	515 (76)	159 (24)
Affectionate support		
Low (<5)	471 (79)	126 (21)
High (≥5)	643 (78)	177 (22)
Tangible support		
Low (<9)	496 (79)	128 (21)
High (≥9)	619 (78)	174 (22)
Positive social interactions		
Low (<9)	618 (80)	153 (20)
High (≥9)	495 (77)	150 (23)
Glasgow stroke outcome scale^f^		
Good recovery	623 (83)	125 (17)
Moderately disabled	327 (76)	102 (24)
Severely disabled	169 (68)	78 (32)
Oxfordshire infarction classification		
Total anterior circulation	24 (69)	11 (31)
Partial anterior circulation	476 (75)	161 (25)
Lacunar	345 (84)	66 (16)
Posterior circulation	177 (78)	49 (22)
Unknown	97 (84)	18 (16)

a Treated for diabetes mellitus (pills or shots).

b Treated for hypertension or blood pressure ≥140/90 mm Hg.

c History of high cholesterol requiring pills.

d Active hormone therapy or current postmenopausal hormone use at Women's Health Initiative enrollment (unopposed estrogen and estrogen with progesterone).

e Episodes/week of moderate to strenuous physical activity ≥20 minutes duration.

f Glasgow stroke outcome scale scoring where: good recovery=able to return to work or school; moderate disability=able to live independently, unable to return to work or school; severe disability=able to follow commands, unable to live independently.

Among the 1424 women in the primary analytic sample, the median time to ischemic stroke after enrollment was 8.7 years (range=16 days–16 years). Median number of depression assessments per participant during our defined follow‐up (between 30 days and 5 years after stroke) was 3 (range=1–8). The median completion of study activity was 100%. In comparing study activity completion by *prestroke* and *postroke* depression status as well as by psychosocial variables, there was no evidence of informative differential missingness. The median time from incident ischemic stroke to first depression assessment was 18.6 months (range=1–54.8 months). Of the 305 women who developed NPSD, median time from stroke to NPSD was 16.0 months (range=33 days–5 years).

Controlling for age at stroke and race, women with good recovery and a moderate level of optimism had a 0.40‐fold reduction in the odds of developing NPSD compared to those with low levels of optimism (*P*<0.01). Among those with good recovery, a low level of adverse life events was also associated with a decreased odds of NPSD (compared to no events, OR, 0.52; 95% CI, 0.27–0.99; *P*=0.05). Associations persisted between all 3 models after additionally controlling for the demographic, behavioral, health‐related, and comorbid potential confounders (Table 2). Among stroke survivors with moderate or severe disability, there were increased odds of developing NPSD with high levels of emotional/information (OR, 1.94; 95% CI, 1.25–2.99; *P*<0.01), tangible (OR, 1.57; 95% CI, 1.02–2.44; *P*=0.04), and positive social interaction support (OR, 1.68; 95% CI, 1.09–2.56; *P*=0.02) after adjusting for all covariates. No other psychosocial factors were significantly associated with NPSD.

**Table 2:** Odds of New‐Onset Depressive Symptoms Within 5 Years After Stroke in Relation to Prestroke Psychosocial Factors^a^

	Good Recovery^b^				Moderate or Severe Disability^c^			
	Cases/Controls	Odds Ratio	95% CI	*P* Value	Cases/Controls	Odds Ratio	95% CI	*P* Value
Optimism	95/456				131/346			
Low (<22)	29/98	1			36/85	1		
Moderate (22–23)^d^	12/113^d^	0.28^d^	0.13 to 0.62^d^	<0.01^d^	33/70	1.10	0.61 to 2.00	0.76
High (24–25)	26/125	0.66	0.35 to 1.27	0.21	28/93	0.65	0.35 to 1.21	0.17
Very high (≥26)	28/120	0.70	0.36 to 1.34	0.28	34/98	0.85	0.47 to 1.53	0.59
Hostility	93/449				129/343			
Low (0–1)	25/123	1			33/89	1		
Moderate (2–3)	19/118	0.85	0.43 to 1.68	0.64	31/89	0.87	0.48 to 1.58	0.64
High (4–5)	29/111	1.25	0.66 to 2.36	0.49	33/83	1.01	0.55 to 1.85	0.97
Very high (≥6)	20/97	1.10	0.54 to 2.23	0.79	32/82	1.09	0.59 to 2.00	0.78
Adverse life events	96/459				129/348			
None	35/136	1			37/99	1		
Low (1–2)^d^	18/128^d^	0.51^d^	0.26 to 0.97^d^	0.04^d^	35/106	0.82	0.47 to 1.45	0.5
Moderate (3–4)	26/108	0.85	0.46 to 1.55	0.59	30/81	1.00	0.56 to 1.82	0.99
High (≥5)	17/87	0.75	0.38 to 1.49	0.41	27/62	1.15	0.61 to 2.17	0.66
Overall social support	96/454				130/342			
Low (<33)	23/99	1			32/87	1		
Moderate (33–37)	17/116	0.57	0.27 to 1.18	0.13	23/88	0.71	0.37 to 1.35	0.29
High (38–42)	33/113	1.23	0.65 to 2.33	0.52	29/75	1.03	0.56 to 1.92	0.92
Very high (≥43)	23/126	0.67	0.34 to 1.32	0.25	46/92	1.52	0.85 to 2.71	0.15
Emotional/informational support	95/455				129/346			
Low (<17)	44/240	1			60/206	1		
High (≥17)^d^	51/215	1.25	0.78 to 2.00	0.36	69/140^d^	1.94^d^	1.25 to 2.99^d^	<0.01^d^
Affectionate support	96/459				130/348			
Low (<5)	34/183	1			53/157	1		
High (≥5)	62/276	1.26	0.78 to 2.06	0.35	77/191	1.22	0.80 to 1.89	0.36
Tangible support	95/459				129/349			
Low (<9)	42/202	1			49/170	1		
High (≥9)^d^	53/257	0.93	0.58 to 1.49	0.77	80/179^d^	1.58^d^	1.02 to 2.44^d^	0.04^d^
Positive social interactions	96/460				129/346			
Low (<9)	51/247	1			62/209	1		
High (≥9)^d^	45/213	1.00	0.63 to 1.59	0.99	67/137^d^	1.67^d^	1.09 to 2.56^d^	0.02^d^

a Adjusted for age at stroke, race (white vs nonwhite), college education, normalized neighborhood socioeconomic status index, Women's Health Initiative study cohort, obesity, waist circumference, smoking status, alcohol intake, physical activity, treatment of diabetes mellitus, treatment of hypertension or hypertension status, treatment of elevated cholesterol, hormone use at enrollment (unopposed estrogen and estrogen with progesterone), and Oxfordshire classification.

b Good recovery as defined by Glasgow stroke outcome scale scoring where: good recovery=able to return to work or school.

c Moderate or severe disability as defined by Glasgow stroke outcome scale scoring where: moderate disability=able to live independently, unable to return to work or school; severe disability=able to follow commands, unable to live independently.

d *P*<0.05.

In sensitivity analyses to address the potential for misclassification bias, when NPSD cases were defined only by the Burnam depression screen, the moderate level of optimism remained significantly associated with reduced odds of NPSD in women with good recovery from stroke across all models (in fully adjusted model, OR, 0.06; 95% CI, 0.01–0.60; *P*=0.01) (Table 3). In post hoc models including women with depression during the 5 years prior to the stroke, 28% (544/1970) had depression after ischemic stroke and the moderate level of optimism was significantly associated with reduced odds of poststroke depression as well.

**Table 3:** Odds of New‐Onset Depressive Symptoms Defined by Burnam Scores Within 5 Years After Stroke in Relation to Prestroke Psychosocial Factors^a^

	Good Recovery^b^				Moderate or Severe Disability^c^			
	Cases/Controls	Odds Ratio	95% CI	*P* Value	Cases/Controls	Odds Ratio	95% CI	*P* Value
Optimism	21/312							
Low (<22)	9/68	1						
Moderate (22–23)^d^	1/78^d^	0.06^d^	0.01 to 0.60^d^	0.02^d^				
High (24–25)	6/84	0.54	0.14 to 2.04	0.36				
Very high (≥26)	5/82	0.41	0.09 to 1.77	0.23				
Adverse life events	22/315							
None	5/91	1						
Low (1–2)	7/81	1.40	0.36 to 5.45	0.63				
Moderate (3–4)	4/78	0.65	0.13 to 3.12	0.59				
High (≥5)	6/65	1.01	0.23 to 4.32	0.99				
Emotional/informational support					31/265			
Low (<17)					17/148	1		
High (≥17)					14/117	1.45	0.60 to 3.47	0.41
Tangible support					31/267			
Low (<9)					16/129	1		
High (≥9)^d^					15/138	0.88	0.38 to 2.03	0.76
Positive social interactions					31/265			
Low (<9)					15/154	1		
High (≥9)^d^					16/111	1.75	0.75 to 4.09	0.20

a Adjusted for age at stroke, race (white vs nonwhite), college education, normalized neighborhood socioeconomic status index, Women's Health Initiative study cohort, obesity, waist circumference, smoking status, alcohol intake, physical activity, treatment of diabetes mellitus, treatment of hypertension or hypertension status, treatment of elevated cholesterol, hormone use at enrollment (unopposed estrogen and estrogen with progesterone), and Oxfordshire classification.

b Good recovery as defined by Glasgow stroke outcome scale scoring where: good recovery=able to return to work or school.

c Moderate or severe disability as defined by Glasgow stroke outcome scale scoring where: moderate disability=able to live independently, unable to return to work or school; severe disability=able to follow commands, unable to live independently.

d *P*<0.05.

## Discussion

In this large multicenter prospective and longitudinal cohort, we examined the occurrence and psychosocial correlates of new‐onset depression within 5 years after ischemic stroke among postmenopausal women. Although 3 previous studies have also examined the relationship between psychosocial factors and depression after stroke,^16^, ^17^, ^18^ a major innovation of the current study was the use of the largest prospective study of health associations with comprehensively measured psychosocial factors in postmenopausal women to date and overcame prior sample size limitations by almost 3‐fold. Three major findings emerged from this study. First, new‐onset poststroke depression was common in women, occurring in about 1 out of every 5 participants. Second, among women with strokes of low severity (ie, able to return to work or school), a moderate level of optimism and a low number of adverse life events were independently protective against NPSD. Although both factors remained associated after adjusting for multiple confounders, only moderate optimism emerged as significant in sensitivity analyses. Third, among women with moderate or severe disability, some subtypes of social support were independently associated with *increased* odds of NPSD.

To our knowledge, the current study is the first to find a protective association between moderate optimism or low number of adverse life events and development of poststroke depression. Prior studies in smaller hospital‐ and rehabilitation‐based cohorts identified social isolation and social support after stroke as protective psychosocial factors for poststroke depression.^10^ These and other studies also identified coping through avoidance, limited ability to cope through finding meaning,^18^ and the perception of lacking social support, particularly from a spouse caregiver,^16^ as higher‐risk psychosocial factors for poststroke depression. Because the protective effect sizes of psychosocial factors such as social support are often larger in men, it might be possible to cautiously extrapolate these findings to both sexes, though the actual effect in men would ideally be assessed in future research. However, the observed association between specific subtypes of social support and *increased* NPSD risk among women with moderate or severe disability from their stroke is consistent with prior evidence about the relationship between social support and women's health, which might be a result of (1) the unclear effect of *change* in social support among women^18^ and (2) typically very small social support effect sizes for women because of higher complexity in social network reciprocity and patient/caregiver roles for women (ie, women receiving social support may be providing in return equal or greater amounts of social support to a larger social network, thus higher reported levels of social support might be associated with increased allostatic load and adverse health effects).^42^

Unlike a prior study showing that patients with persistent negative affect after stroke had a 4.6‐fold higher risk of developing poststroke depression compared to those with low negative affect scores regardless of lesion location,^17^ our findings do not solely represent the opposite or absence of depression given independence between positive and negative emotions.^43^ Moderate optimism may independently reduce risk of depression after stroke through several potential behavioral, psychological, and physiological mechanisms. For example, individuals with high levels of pessimism (negative future expectations) or low levels of optimism, independent of socioeconomic status, are more likely to participate in adverse health behaviors that may increase the risk for depression, such as cigarette smoking, poor diet, physical inactivity, and substance abuse.^44^ There are also associations between optimism and both psychological and physiological stress (eg, increased markers of chronic inflammation and increased activity of the neuroendocrine system).^45^

Results from observational studies need to be interpreted in the context of their limitations. First, although we were able to adjust for many important covariates, we were unable to account for all potential confounding or reverse causality bias that might explain results (eg, family history of depression or infarct laterality). Second, the generalizability of results may be limited given our sample of largely white (non‐Hispanic) women with high level of education (37% with college degree or higher) and low frequency of some risk factors (eg, 7% with prevalent diabetes mellitus). Third, there are some inherent challenges in defining poststroke depression. For example, if depressed participants were less responsive to study activities in the 5 years after stroke, there would be potential for misclassification bias. To address this possibility, we assessed for differences in participation by depression status before and after stroke as well as by psychosocial exposure variables and found no significant evidence for informative differential missingness. Conversely, using antidepressant initiation as an adjunct method of defining cases may overclassify depression given that antidepressants can be prescribed for indications outside of depression, such as anxiety. Since 2011, antidepressants have also been used for possible benefit in poststroke motor recovery,^46^ though this trend is unlikely to affect our results given that our study's incident ischemic strokes occurred prior to 2010. We address this limitation by conducting a sensitivity analysis where the Burnam depression screen was the only criterion used to define cases, which supported our initial finding for an association between a moderate level of optimism and NPSD. While post hoc tests add confidence to our findings, exploratory analyses should be replicated in other cohorts to confirm validity of results.

Determining the role of psychosocial factors in preventing depression after stroke is crucial, yet it is not possible to clearly establish a causal relationship between optimism and risk of poststroke depression from observational studies alone. Our results and the findings of extant studies may reflect residual confounding from the inability to accurately measure and adequately adjust for factors such as health service utilization, longstanding psychiatric symptoms, and additional social and environmental exposures that are challenging to capture. While optimism is a potentially modifiable psychosocial factor,^47^ our results may also reflect reverse causality bias whereby lifestyle choices or health‐related behaviors may have determined levels of positive attributes^48^ or the initial risk of stroke.^49^ There is even some evidence to suggest that the benefits from specifically moderate optimism may be partially determined by neurobiological differences.^50^ These complex relationships must be investigated further with techniques for causal inference in observational studies (such as Mendelian randomization),^51^ more detailed social network‐level characteristics (such as dyadic and supradyadic effects),^52^ or inclusion of novel dynamic measures of social and behavioral functioning (such as digital phenotyping).^53^

## Conclusion

Though rarely studied in poststroke depression, understanding how prestroke psychosocial factors, such as optimism, are associated with the risk for developing new‐onset depression after ischemic stroke may ultimately aid in improving the identification of stroke survivors at highest risk for depression who may benefit from early prevention through a targeted intervention strategy in the acute to subacute poststroke period.

## Appendix

### Abbreviated List of WHI Investigators

*Program Office:* (National Heart, Lung, and Blood Institute, Bethesda, MD) Jacques Rossouw, Shari Ludlam, Dale Burwen, Joan McGowan, Leslie Ford, and Nancy Geller.

*Clinical Coordinating Center:* Clinical Coordinating Center: (Fred Hutchinson Cancer Research Center, Seattle, WA) Garnet Anderson, Ross Prentice, Andrea LaCroix, and Charles Kooperberg.

*Investigators and Academic Centers:* (Brigham and Women's Hospital, Harvard Medical School, Boston, MA) JoAnn E. Manson; (MedStar Health Research Institute/Howard University, Washington, DC) Barbara V. Howard; (Stanford Prevention Research Center, Stanford, CA) Marcia L. Stefanick; (The Ohio State University, Columbus, OH) Rebecca Jackson; (University of Arizona, Tucson/Phoenix, AZ) Cynthia A. Thomson; (University at Buffalo, Buffalo, NY) Jean Wactawski‐Wende; (University of Florida, Gainesville/Jacksonville, FL) Marian Limacher; (University of Iowa, Iowa City/Davenport, IA) Robert Wallace; (University of Pittsburgh, Pittsburgh, PA) Lewis Kuller; (Wake Forest University School of Medicine, Winston‐Salem, NC) Sally Shumaker.

*Women's Health Initiative Memory Study:* (Wake Forest University School of Medicine, Winston‐Salem, NC) Sally Shumaker.

## Sources of Funding

The WHI program is funded by the National Heart, Lung, and Blood Institute, National Institutes of Health, U.S. Department of Health and Human Services through contracts HHSN268201600018C, HHSN268201600001C, HHSN268201600002C, HHSN268201600003C, and HHSN268201600004C. Research reported in this publication was supported by the Schwamm Marriott Clinical Care Research Fellowship Program and National Institutes of Health training grant T32NS048005 (Salinas) as well as the National Institute of Mental Health under award number K01MH102403 (Dunn). The content is solely the responsibility of the authors and does not necessarily represent the official views of the National Institutes of Health.

## Disclosures

None.
